# Editorial: Current Status and Future Challenges of Biobank Data Analysis

**DOI:** 10.3389/fgene.2022.882611

**Published:** 2022-04-14

**Authors:** Tzu-Pin Lu, Yoichiro Kamatani, Gillian Belbin, Taesung Park, Chuhsing Kate Hsiao

**Affiliations:** ^1^ Department of Public Health, College of Public Health, Institute of Epidemiology and Preventive Medicine, National Taiwan University, Taipei, Taiwan; ^2^ Department of Computational Biology and Medical Sciences, Graduate School of Frontier Sciences, The University of Tokyo, Tokyo, Japan; ^3^ Institute of Genomic Health, Icahn School of Medicine at Mount Sinai, New York, NY, United States; ^4^ Department of Statistics, Seoul National University, Seoul, South Korea

**Keywords:** biobank data analysis, GWAS, integrative analysis, personalized medicine, statistical genetics

Global health care systems are severely strained under the burden of aging populations, which are prone to enhanced incidence of chronic diseases. Biobanks help manage the population-wide disease burden by providing resources for scientific and medical research, allowing improved public health and individual patient care (Harris et al., 2012; Jacobs et al., 2018). Biobanks are the result of the combination of emerging novel technologies, well-catalogued human biological samples, and corresponding clinical and research data (Harris et al., 2012). The last 2 decades have witnessed the emergence of several biobanks around the world. Their primary aims are to uncover the genetic etiology of various chronic illnesses and to study the interaction of ethnicity with the underlying clinico-pathology and genetics of individuals across different populations (Sanner and Frazier, 2007).

A wide spectrum of phenotypic information is available in biobanks, including diagnoses, risk factors, physical and metabolic parameters, clinical information, as well as data on behavioral and social factors (Harris et al., 2012; Jacobs et al., 2018; Coppola et al., 2019). Single nucleotide polymorphism (SNP) genotyping arrays allow genome-wide association studies (GWASs) and polygenic risk score (PRS) development (Jimmy Juang et al., 2020) or phenome-wide association studies (PheWASs) (Liu and Crawford, 2022). Moreover, findings from GWASs can be utilized to conduct genetic tests on cohorts (Yamamoto et al., 2017). Biobanks require strict quality standards, which has led to the discovery of many new diagnostic and prognostic markers and a better pathophysiological understanding of disease development (Jacobs et al., 2018).

Biobanks provide high-dimensional data with large sample sizes, allowing gains in statistical power towards identification of novel genetic findings. However, the sheer amount of data they contain presents a greater computational burden that needs to be dealt with carefully. Another issue associated with traditional GWASs is the existence of linkage disequilibrium that leads to non-causal genetic markers. Additionally, for weak signals (minor allele frequency (MAF) ≤0.01), existing statistical models may not be powerful enough to detect putative rare variants. Hence, before conducting analyses and building models, it becomes imperative to reduce the data dimension while retaining the essential information of the original data (Sakaue et al., 2020). This is especially relevant for studies that aim to account for missing heritability, due to the modest effect of GWAS-reported loci on disease risk of complex traits, through genetic interactions (gene-gene, gene-environment). Prospective interactions exponentially increase with the increase in total genetic loci, leading to a number of challenges associated with high-dimensional data, as otherwise known as the curse of dimensionality (Chattopadhyay and Lu, 2019). Therefore, robust state-of-the-art computational techniques are required in order to identify and validate genetic interactions, eventually leading to a body of evidence that can explain a part of the current gap in heritability of complex traits and diseases.

High-throughput techniques have made available to researchers the genomic information from multiple platforms, such as DNA single nucleotide variants (SNVs), copy number variations (CNVs), and DNA methylation from SNP microarrays and/or short- or long-read next-generation sequencing platforms. Genetic susceptibility to a specific disease can be gauged better if combined multi-omics studies can be conducted for each patient/individual (Curtis et al., 2012; Brucker et al., 2020). Analysis of a single type of -omic data is limited by correlations primarily providing reactive signals rather than real causal ones, while integration of multiple -omics data types potentially may identify real causal changes that elucidate disease etiology or treatment targets (Hasin et al., 2017). Such findings can subsequently be tested for confirmation in molecular studies.

This Research Topic aimed to bring together studies that can showcase the potential and wealth of information that biobanks hold by providing a comprehensive overview of the current advances in scientific and clinical knowledge derived from biobank data analysis. Primarily, it aimed to compile algorithms and statistical models that report novel findings for different phenotypes, perform integrated analyses of multi-omics data in health and disease, and report sophisticated bioinformatics and statistical techniques with the aim of establishing an association between the genetic profiles and biological phenotypes. The second goal was to weigh the various difficulties and challenges posed involved in high-dimensional biobank data analysis. To this end, five studies were published under this Research Topic, as described in the following sections. Four studies were conducted on subjects from the Taiwan Biobank (TWB) (Wei et al., 2021), and the fifth was a review discussing the challenges related to biobank data analysis.

The TWB was established in 2012, with the aim of creating a population-based cohort of 200,000 adults, recruited at regular time intervals, with no cancer diagnosis at the time of enrollment (Juang et al., 2021). The general population of Taiwan constitutes individuals of Han-Chinese ancestry who immigrated from various provinces of China and local Taiwanese aboriginals. For genotype imputation purposes, the TWB additionally offers a reference panel created from whole-genome sequencing data from 1,445 early recruited participants. Furthermore, it offers two customized SNP genotyping arrays. Being the largest publicly available genetic database of individuals with East Asian ancestry, it helps document population-specific risk variants to improve the clinical care of the participants.

A study by Chen et al. described the association of a much-reported variant *ABCG2* rs2231142 and body mass index (BMI) with the risk of incidence of hyperuricemia (HUA), a major risk factor for gout, in East Asian populations. HUA is associated with various comorbidities, including obesity, hypertension, type 2 diabetes, hyperlipidemia, cardiovascular diseases, chronic kidney diseases, stroke, osteoporosis, erectile dysfunction, and obstructive sleep apnea, and has been reported as an independent predictor of premature mortality (Vincent et al., 2017; Singh and Cleveland, 2019; Huang et al., 2020). They conducted their study on 4,228 HUA patients from the TWB and reported a higher risk of HUA in association with either the “risk T” allele of *ABCG2* rs2231142 (TT or TG genotype) or higher BMI for both men and women. They further established the association of strong genetic-environmental (GxE) interaction with very high risk of HUA. Based on their findings, they recommended controlling body weight (i.e., lowering BMI) for patients with high risk of HUA carrying the *ABCG2* rs2231142 risk T allele.

Three studies were published under this Research Topic that conducted genomic analysis through proposed scalable methodologies to improve the relevance, utility and interpretability of the reported findings. One was by Chi et al., who proposed SEAGLE, a scalable exact algorithm for large-scale set-based gene-environment (G × E) tests on continuous traits, and applied it to subjects from the TWB. SEAGLE deploys matrix computations to calculate variance-component test statistics and p-values of G × E interactions. It requires no additional assumptions or approximations and is computationally efficient, with the ability to accommodate sample sizes up to the order of 10^5^, thereby eliminating the requirement for high performing computing resources. Extensive simulation studies under different scenarios and assumptions were conducted to establish its scalability and comparable power and type I error rates.

The second study was by Yu et al., who proposed an integrative co-localization (INCO) approach for combining more than one type of -omic data. SNVs and CNVs from the same genomic unit were utilized for obtaining their concurrent effect and dealing with the sparsity of rare variations. Traditional integrated analyses of multi-omics data usually analyze each type of data separately, after which a naïve union or intersection analysis of significant findings is conducted to identify candidate genes. Such approaches may fall behind in identifying causal variants for traits that are a result of the concurrent effect of both the omics levels. INCO is a hybrid approach that conducts a screening procedure at the gene level, followed by modeling a concurrent effect from both levels of data, irrespective of whether each of them has a marginal association with the trait. Finally, it focuses on narrower genetic regions for bypassing the sparsity effect due to rare variants. Yu et al. conducted comprehensive simulations to demonstrate the scalability of their approach under different assumptions and scenarios and then applied their method to the study of subjects from the TWB, specifically their low-density lipoprotein cholesterol and triglyceride levels. They reported a potentially novel association of the *VNN2* gene, which is a protein coding gene involved in cell migration and fatty acid metabolism.

Finally, the third study was reported by Sun et al. and demonstrated that a novel quality control procedure can improve the accuracy of rare variant calling in SNP arrays. Detection of rare variants through genomic association studies still remains a challenge for various reasons—noisy signals and batch effects to name a few—and improvement in the genotyping quality may be an avenue for better clinical applications. Analyzing a custom Axiom array of data consisting of 267,247 rare variants obtained from 43,433 individuals in the TWB, an advanced normalization adjustment was adopted to prevent false calls caused by splitting the cluster, and a rare het adjustment was employed to lower false calls of rare variants. The concordance of the MAF for the called variants was measured by comparing it with that of the allelic frequencies from array data. Finally, genotyping results were used to detect familial hypercholesterolemia, thrombophilia, and maturity-onset diabetes of the young to assess the performance of their proposed procedure in disease screening. All heterozygous calls were verified by Sanger sequencing or qPCR, and the positive predictive value of each step was reported with an increase of up to 100%. Findings from this study demonstrated that correctly conducted genotype calling of rare variants could potentially be a solution for pathogenic variant detection through SNP arrays.

Lastly, a review was published by Bi and Lee, where they discussed challenges in multi-omics data analysis with the aim of aiding statisticians, epidemiologists, and other medical scientists in dealing with biobank-level data. They described in detail the current and future statistical and computational roadblocks that researchers stumble upon while performing GWASs (single point association or multilevel epistatic association) and PheWASs on large-scale biobank data; summarized recently developed scalable and robust regression approaches; and introduced Phewebs and some phenome-wide analysis results at the variant, gene, and pathway levels. They further outlined the need for more advanced methods and tools for handling future challenges and furnished comprehensive information for statisticians to obtain an up-to-date understanding of the tools and technologies at hand.

Based on these published studies in this Research Topic, we discussed some of the difficulties in analyzing massive amounts of genetic data. In addition to the methodological issues, another crucial challenge now is to combine biobanks worldwide for comprehensive studies to improve the equity of obtaining genetics data in human genome research. Since genomic research findings are often translated into genetic testing, disease diagnosis, and therapeutic solutions, especially in the era of personalized medicine, it is important that the scientific conclusions are not drawn from a biased sample to enhance population health globally (Harris and Sulston, 2004; Editorial, 2021). One way to do this would be to have a collective effort from researchers conducting studies with genetic databases such as biobanks around the world, so that diversity can be achieved. Several initiatives have started to reach this goal (The H3Africa Consortium, 2014; GenomeAsia100K Consortium, 2019; Robine and Varmus, 2021). We have not covered this in the current Research Topic, but we hope there will be one focusing on this issue in the near future.

In summary, biobanks play a central role in elucidating disease etiology and promoting public health. Incorporating biological, clinical, and genetic information into multi-omics analysis protocols is crucial to this end. Efficient use of big data by biobanks, both retrospectively and prospectively, would accelerate the implementation of preventive measures, optimized treatments, and personalized healthcare.
